# Research on Resilient Modulus Prediction Model and Equivalence Analysis for Polymer Reinforced Subgrade Soil under Dry–Wet Cycle

**DOI:** 10.3390/polym15204187

**Published:** 2023-10-23

**Authors:** Yingcheng Luan, Wei Lu, Kun Fu

**Affiliations:** 1 Research Center of Geotechnical and Structural Engineering, Shandong University, Jinan 250061, China; 2 School of Transportation, Southeast University, 2# Southeast University Road, Jiangning District, Nanjing 210096, China

**Keywords:** polymer reinforced subgrade soil, dry-wet cycle, resilient modulus, pavement structure, finite element analysis, moisture content

## Abstract

The subgrade soil of asphalt pavement is significantly susceptible to changes in moisture content, and therefore many projects introduce polymer-based reinforcement to ensure soil performance. This paper aims to incorporate a variable representing the dry–wet cycle into the prediction model of resilient modulus of polymer reinforced soil. The polymer adopted is a self-developed subgrade soil solidification material consisting of sodium dodecyl sulfate and polyvinyl oxide. The current resilient modulus prediction model is improved, notably involving the effects of the dry–wet cycle. Combined with finite element method (FEM) analysis, the actual stress state of pavement and the coupling effect of dry–wet cycle and vehicle load on the resilient modulus are studied. The deterioration in resilient modulus with the variation in seasonal climate and load response is also investigated. Results show that the deviator stress is negatively correlated with the resilient modulus while the bulk stress has a linearly positive relation. The decreasing rate at low deviator stress is larger than that at the high level. Moreover, the dry–wet cycle can reduce the resilient modulus and the reducing amplitude is the largest at the first dry–wet cycle. FEM analysis shows that the middle position of the subgrade slope has the largest initial resilient modulus with decreasing amplitude in the first year of dry–wet cycles, while the upper position shows a smaller change. The variation in resilient modulus is closely related to the changes in cumulative volumetric water content. Considering that different positions of subgrade bear the external vehicle load, the equivalent resilient modulus is more realistic for guiding the subgrade design.

## 1. Introduction

The subgrade is the foundation of the pavement structure and bears the loads from the weight of vehicles and pavement structure. A change in bearing capacity will greatly affect the stability and service life of a pavement structure [1,2], and the bearing capacity is commonly represented by the resilient modulus, which is affected by the dry–wet cycles, resulting in the reduction in strength of the subgrade soil [3,4]. For this reason, many projects introduce polymer-based reinforcement to ensure soil performance.

The physical condition of optimal moisture content (OMC) is usually adopted to achieve the maximum compaction density and a better engineering performance [5]. This indicates that variation in seasonal moisture content and suction due to exposure to the external environment have significant effects on the mechanical performance of subgrade soil, especially for the resilient modulus [6,7]. Many models have been proposed to predict the resilient modulus of the subgrade soil and other bases of unbonded aggregate materials, which is generally in non-linear form and influenced by many factors, such as stress state, hydraulic hysteresis, physical microstructure, etc. [8,9,10]. In order to study the effect of moisture content on the resilient modulus, in a prediction model employed to study the direct effect of moisture, suction was considered to be significant, and adopted as an independent term [3,11,12]; suction also has an influence on confining or shear stress [13,14,15]. In addition, the soil specimens that suffered from drying and wetting have a larger permanent deformation and lower resilient modulus compared to those specimens that are not subjected to the fluctuation in moisture content [16]. Atmospheric rainfall changes periodically, resulting in the annual occurrence of subgrade settlement and deformation, which endangers the highway capacity and driving safety [17]. Therefore, the effects of the dry–wet cycle on the resilient modulus should be included in a more realistic prediction model.

Due to the fact the resilient modulus changes with the variation in seasonal rainfall and the experimental conditions are limited, large differences in environmental factors and stress conditions exist between the experimental and actual pavement conditions, indicating that the experiment cannot represent the actual pavement service condition. This limitation can be broken through by conducting a finite element method (FEM) analysis and the actual state of the pavement structure can thus be better characterized. Hu et al. have carried out a slope stability analysis using ABAQUS FEM software while considering the dry–wet cycle [18]. Chen et al. have developed a numerical model to analyze the unsaturated flow in porous media with repeated dry–wet cycles [19]. Yaqi et al. proposed an analytical framework to predict the longitudinal shrinkage crack by incorporating different dry/wet paths into numerical modeling [20]. Other research is mainly concentrated on the effects of the dry–wet cycle on engineering performance. However, limited efforts have been made to explore the coupling effect of traffic load and dry–wet cycles on the resilient modulus of subgrade.

The resilient modulus of unsaturated soil is affected by the stress state, humidity state, and dry–wet cycle. In this paper, the current resilient modulus prediction model is improved, and the effects of dry–wet cycle are included. Based on this constitutive model of resilient modulus, the finite element method (FEM) is adopted to simulate the actual stress state of the pavement and the effects of the dry–wet cycle are studied by introducing the UMAT user-defined subroutine. The deterioration of resilient modulus with the variation in seasonal climate and load response is next investigated, and the resilient modulus of the whole subgrade can be reasonably estimated. This will provide a basis for the design of the soaked subgrade.

## 2. Resilient Modulus Prediction Model

### 2.1. Development of Resilient Modulus Model

The conventional prediction models of resilient modulus depend on bulk stress, or deviatoric stress Refs. [21,22,23], which was proposed by the National Cooperative Highway Research Program (NCHRP) and is used by many researchers (Equation (1)) to study the mechanical response of the unbonded material [24]:

(1) $$E_{y}=q_{1}A_{a}{\left(\frac{C_{1}}{A_{a}}\right)}^{q_{2}}{\left(\frac{D_{oct}}{A_{a}}+1\right)}^{q_{3}}$$

where *C*_1_ is the term of bulk stress; *D_oct_*, the term of deviatoric stress; *A_a_*, the atmospheric pressure; and *q*_1_, *q*_2,_ and *q*_3_, the regression coefficients. This model shows the resilient modulus is dependent on the stress state. However, the unbonded aggregate is also a kind of moisture-dependent material. The saturation degree and matric suction are regarded to be the influencing factors for the resilient modulus and have thus been included in the prediction models. AASHTO employed the saturation degree to characterize the moisture sensitivity, which has been adopted in the MEPDG [25]. Heath et al. incorporated the normalizing matric suction to predict the resilient modulus [26]. Therefore, both the stress state and moisture variation could be considered in the resilient modulus model. However, Sahin et al. have pointed out that the moisture-sensitivity not only depends on the saturation degree but also the matric suction, and both of them were therefore included in a new model as shown in Equation (2) [13]:

(2) $$E_{y}=q_{1}A_{a}{\left(\frac{C_{1}-3\phi s\omega }{A_{a}}\right)}^{q_{2}}{\left(\frac{D_{oct}}{A_{a}}\right)}^{q_{3}}$$

where *ϕ* is the volumetric water content; *ω*, the matric suction related to the water content; and *s*, the saturation parameter. The matric suction can be obtained by the soil–water characteristic curve (SWCC). The SWCC of a targeted soil could be obtained by the model proposed as shown in Equation (3) [27]:

(3) $$\phi =\frac{\phi_{s}}{{\left[\ln\left(e+{\left(\frac{\omega }{\alpha A_{a}}\right)}^{\beta }\right)\right]}^{\gamma }}$$

where *ϕ_s_* is the saturated volumetric water content; and *α*, *β*, and *γ*, the fitting coefficients.

### 2.2. Resilient Modulus with Dry–Wet Cycle

By contrast, there are limited studies that focus on the effect of drying–wetting cycles on the resilient modulus. Combined with SWCC, the cumulative increment of volumetric water content is used to characterize the effects of the drying–wetting cycle. The volumetric water content is calculated according to Equation (4), while the increment form is shown in Equation (5) and is represented by the absolute value. The cumulative increment of the volumetric water content has an increasing trend with the increase in cycle number and amplitude. Since a constant amplitude of the dry–wet cycle is adopted in this paper, the expression can be expressed as Equation (6).

(4) $$\phi =\frac{V_{w}}{V}=\frac{m_{w}/\rho_{w}}{1+e}=\frac{w\cdot m_{s}}{\left(1+e\right)\cdot \rho_{w}}=\frac{w\cdot G_{s}}{1+e}$$

In the above equation, *V_w_* is the volume of water; *V*, the volume of dry soil particles; *m_w_*, the moisture content; *ρ_w_*, the density of water; *e*, the void ratio; *G_s_*, the specific gravity of soil particles; and *w*, the rate of water content.

(5) $$\sum d\phi =\int_{0}^{N}\frac{dw\cdot G_{s}}{1+e}dN$$

(6) $$\sum d\phi =\frac{N\cdot dw}{1+e}\cdot G_{s}$$

In the above equations, *N* is the number of cycles; and *dw*, the variation in rate of water content.

Two aspects are considered when constructing the prediction model: (1) the effects from the drying–wetting cycle should be considered, that is, the resilient modulus shows a converse relation with the times and amplitudes of the drying–wetting cycle; (2) the cumulative volumetric water content and the matric suction in the SWCC should be involved. The prediction models are presented in the form of power function and exponential function as shown in Equations (7) and (8). The final form is then determined by the experimental data fitting results.

(7) $$E_{y}=q_{1}A_{a}{\left(\frac{C_{1}-3\phi s\omega }{A_{a}}\right)}^{q_{2}}{\left(\frac{D_{oct}}{A_{a}}+1\right)}^{q_{3}}\left(1+q_{4}{\left(\int_{0}^{N}\frac{dw\cdot G_{s}}{1+e}dN\right)}^{q_{5}}\right)$$

(8) $$E_{y}=q_{1}A_{a}{\left(\frac{C_{1}-3\phi s\omega }{A_{a}}\right)}^{q_{2}}{\left(\frac{D_{oct}}{A_{a}}+1\right)}^{q_{3}}\left(1+q_{4}\exp\left(q_{5}\int_{0}^{N}\frac{dw\cdot G_{s}}{1+e}dN\right)\right)$$

## 3. Materials and Experiments

### 3.1. Raw Materials and Specimen Preparation

#### 3.1.1. Soil

**Figure 1:**
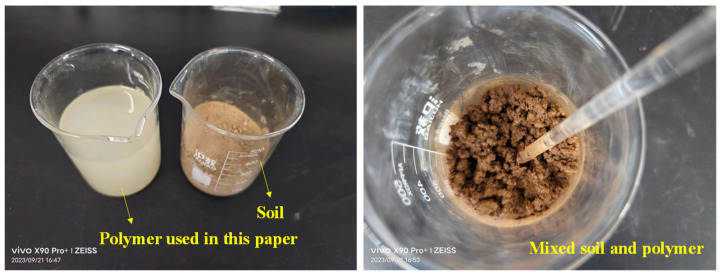
The reinforcement process of soil.

This paper takes a typical polymer-reinforced soft subgrade soil section under dry–wet cycle conditions as an example (Figure 1). During the road construction process, due to the dry–wet cycling effect, the mechanical properties of the soil cannot meet the design requirements, so polymer-based reinforcement agents are introduced as subgrade soil reinforcement materials. The polymer adopted is a self-developed soil solidification material consisting of sodium dodecyl sulfate and polyvinyl oxide. The tested results of the polymer-based reinforcement agent are shown in Table 1.

Due to the introduction of polymer-based reinforcement agents, the properties of reinforced soil are quite different from the original soil. The experimental soil belongs to the clay and comes from a test section in Pukou District, Nanjing. The specific physical properties are shown in Table 2.

#### 3.1.2. Filter Paper Test

**Figure 2:**
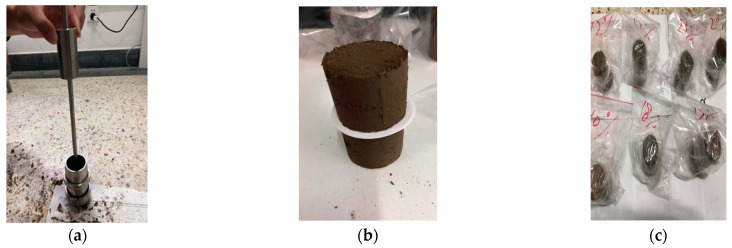
The specimen preparation: (**a**) formation, (**b**) filter paper, (**c**) seal.

The filter paper test was applied to measure the matric suction in accordance with ASTM D5298. A series of moisture contents were tested so as to establish the optimal moisture content: these were 12%, 15%, 17%, 18%, 20%, 23%, and 25%, respectively. The cylindrical specimens were formed with the dimension of Φ 3.91 cm × 8 cm. Three pieces of filter paper for each experiment were prepared and placed between two sections of a soil sample that had been sawn in half. Only the middle filter paper was used for measuring the variation in moisture weight, while the other two were to prevent the pollution of the middle filter paper. The entire sample was then sealed and stored at the temperature of 60 °C for 7 days to ensure a sufficient balance of moisture between the filter paper and the soil sample. The specimen preparation is shown in Figure 2.

### 3.2. Triaxial Test of Dynamic Resilient Modulus with Dry–Wet Cycle

#### 3.2.1. Dry–Wet Cycle

In order to study the effect of drying–wetting cycles on the resilient modulus of compacted soil, a test with different numbers of drying–wetting cycles was carried out. The resilient modulus was then measured. The initial water content of 12% was set as the starting point and the amplitude value was ±2%. Four times of cycle were determined, i.e., 1, 2, 3 and 4. The moisture content first increased from the initial value of 12% to 14%, then the specimens were dried in an oven down to 10%, followed by continuing to add water back up to 12% for a cycle: namely, 12% to 14% to 10% to 12%. The specific implementation was as follows: (1) humidification: the required water content was calculated and injected into the sample by a syringe to reach the target peak. When adding water, the surface of the sample was covered with a filter paper to reduce the erosion of the sample surface, then sealed and moisturized for 24 h; (2) dehumidification: the specimen was placed in the oven for dehumidification after the humidification process was completed; then the specimen was weighed every 10 min to measure the variation in moisture content until it reached 10%, and was then sealed for 24 h.

#### 3.2.2. Dynamic Resilient Modulus

**Figure 3:**
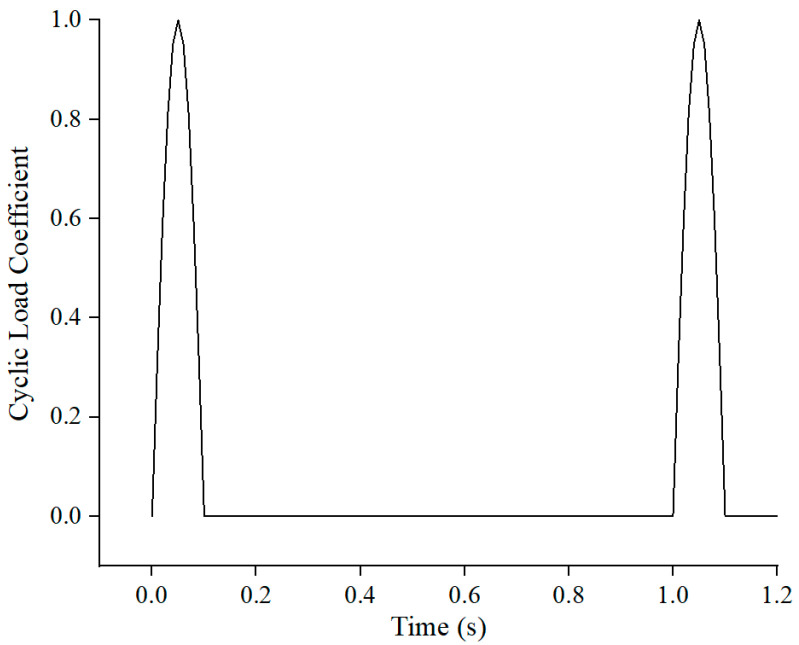
Diagram of cycle load.

**Figure 4:**
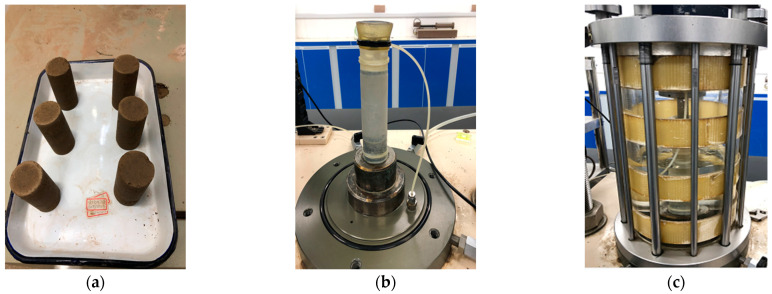
Dynamic triaxial test process: (**a**) specimens, (**b**) sample placement, (**c**) triaxial test.

The GDS unsaturated soil triaxial apparatus can simulate the mechanical response of the soil under dynamic loading. The Haversine wave loading mode was used as it can best reflect the typical stress state inside the road. The period was set as 1.0 s, in which the load pulse was 0.1 s and the rest period was 0.9 s. The pre-loading was performed to eliminate the occurrence of large plastic deformation at the initial stage and to simulate the stress history of subgrade soil. The loading sequence is listed in Table 3, while the dynamic triaxial test process is shown in Figure 3 and the testing process is shown in Figure 4.

The resilient modulus was carried out by AASHTO T-307. For each loading sequence, the data of the last five loading cycles were taken to calculate the dynamic resilient modulus when the strain response became stable. The calculation of *M*_r_ is shown in Equations (9)–(11).

(9) $$\sigma_{d}=\frac{P_{i}}{A}$$

In the above equation, *σ_d_* is the axial stress; *P_i_*, the average amplitude of the last five axial loadings (N); and *A*, the average cross-sectional area of the upper and lower surfaces of the specimen (mm^2^).

(10) $$\varepsilon_{0}=\frac{\Delta_{i}}{l_{0}}$$

In this equation, *ε*_0_ is the recoverable strain (mm/mm); Δ*_i_*, the average axial deformation amplitude of the last five loadings (mm); and *l*_0_, the measurement distance of displacement sensor (mm).

(11) $$M_{r}=\frac{\sigma_{d}}{\varepsilon_{0}}$$

Here, *M_r_* is the dynamic resilient modulus.

## 4. Analysis of Resilient Modulus Model

### 4.1. The Curve of SWCC

**Figure 5:**
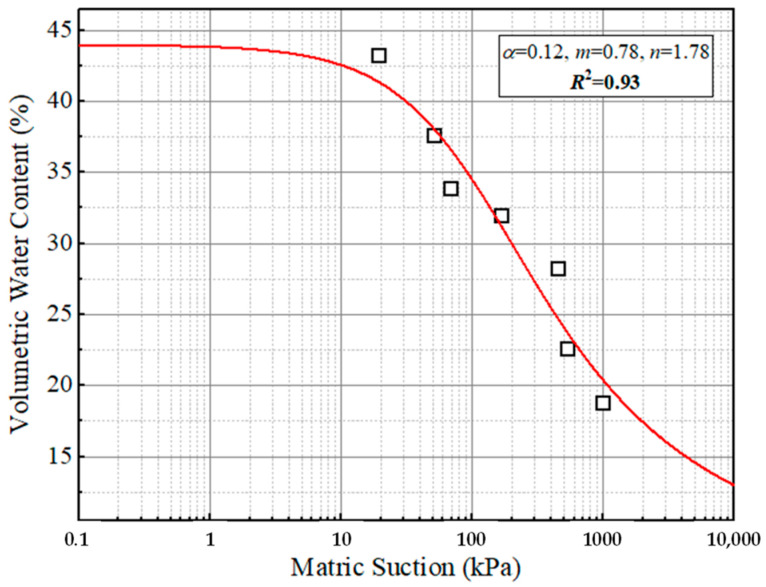
Soil water characteristic curves for soil material.

The SWCC was obtained for the tested soil material according to the testing results of the filter paper test, as shown in Figure 4. The fitting accuracy is 0.93. Where the water content is given, the matric suction will have been determined from Figure 5.

### 4.2. Results of Triaxial Test

#### 4.2.1. Effect of Deviator Stress

**Figure 6:**
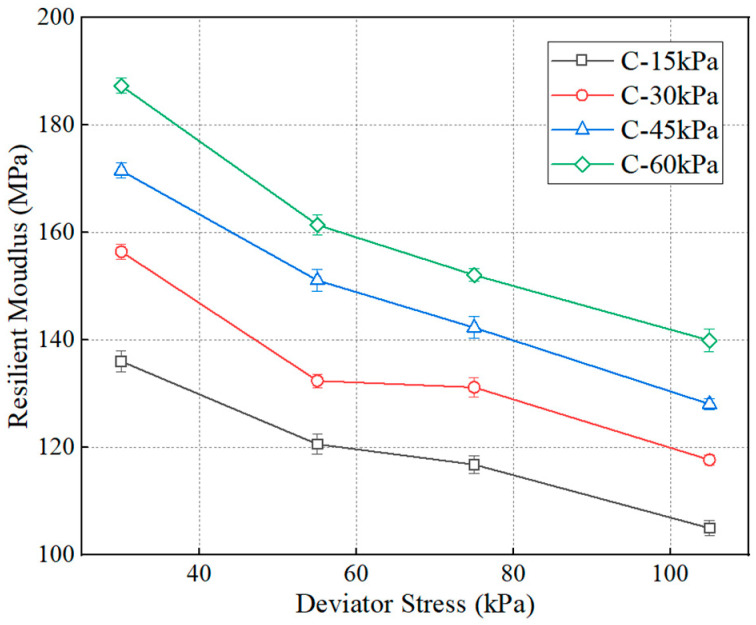
The trend between resilient modulus and deviator stress.

Figure 6 presents the trends between the resilient modulus and the deviator stress for the specimens without dry–set cycle, in which reference to C in the legend is to the abbreviation for confining stress. The resilient modulus has a negative relation to the deviator stress. This may be because higher deviator stress results in a larger vertical strain, and the increment in vertical strain is greater than the increased value of deviator stress, which leads to a reduction in resilient modulus [29]. Taking the group of C-60 kPa as an example, the decreasing rates are 14.1%, 19.0%, and 25.4% when the deviator stress increases from 30 kPa to 55 kPa, 75 kPa, and 105 kPa, respectively. This is consistent with other findings [30,31]. In addition, the decreasing rate has a larger value at low deviator stress while a smaller decreasing amplitude can be found at high deviator stress, indicating that the resilient modulus has a dependence on the deviator stress. This may explain why the vertical compressive strain should be controlled for the subgrade design. The stress state can be calculated once the pavement structure is determined, and the vertical compressive strain is limited to a specified range to meet the requirements of minimum value for resilient modulus.

#### 4.2.2. Effect of Confining Stress

**Figure 7:**
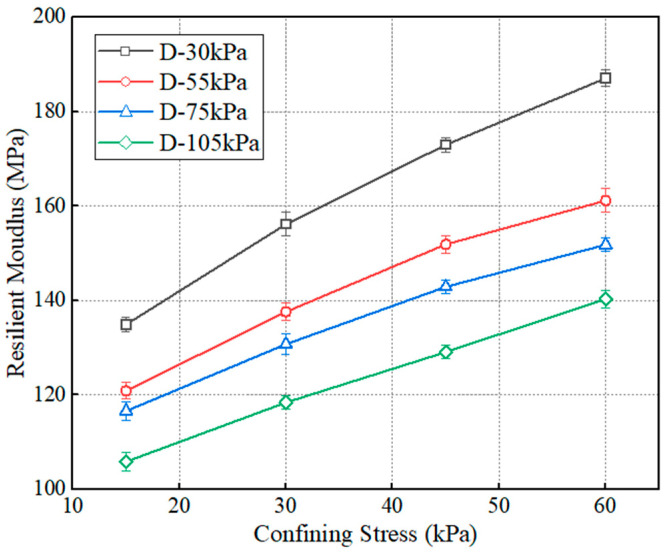
The variation in resilient modulus with confining stress.

Figure 7 shows the variation in resilient modulus with the confining stress: the specimen without dry–set cycle was used. The abbreviation of *D* in the legend represents the deviator stress. The confining stress has a lateral restraint effect on the specimens and promotes a better bearing capacity. Thus, the resilient modulus is linearly positively correlated with the confining pressure. This is in agreement with previous studies [32,33].

#### 4.2.3. Effect of Stress State

**Figure 8:**
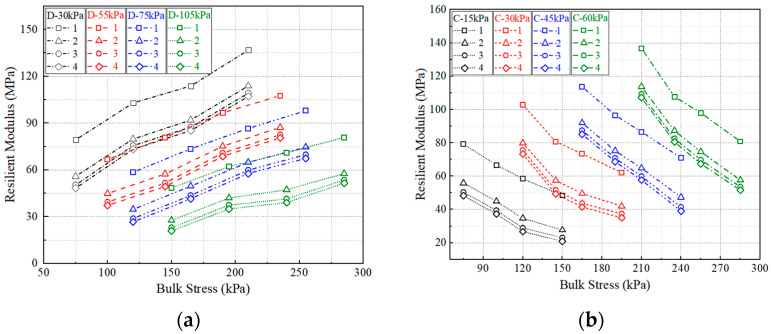
Resilient modulus with the change in stress state: (**a**) deviator stress, (**b**) confining stress.

Figure 8 shows the changes in resilient modulus with the variation in deviator stress and bulk stress. The figures in the legend represent the number of dry–wet cycles. For the same curve in Figure 8a, the deviator stress is fixed, the increasing bulk stress indicates that the confining stress is increased, while the curves in Figure 8b represent the variation in deviator stress. It can be seen that the resilient modulus increases with the confining stress, and has a negative relationship to the deviator stress. Previous studies have found that the deviator stress has a more significant effect than the confining stress for fine-graded soils, especially for clay soil [34]. The deviator stress reflects the shear effect which is prone to resulting in a softening effect for the subgrade soil [32].

#### 4.2.4. Effect of Dry–Wet Cycle Number

**Figure 9:**
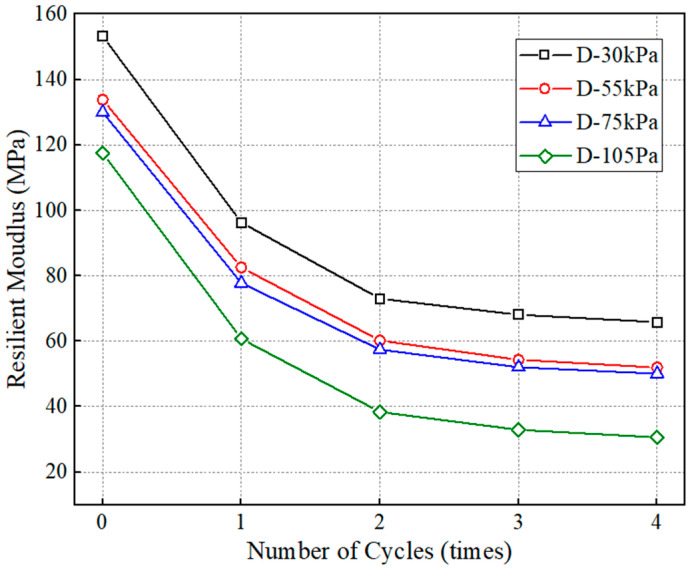
The variation in resilient modulus with number of dry–wet cycles.

The confining stress in the subgrade is generally distributed from 25 kPa to 40 kPa, from which 30 kPa was selected. Figure 9 shows the relations between the variation in resilient modulus and the number of the dry–wet cycle. The effects of the dry–wet cycle can result in a reduction in resilient modulus. The reducing amplitude caused by the first cycle is the largest, at 37%. After that, the reducing rate decreases, and the resilient modulus almost reaches a stable state after four cycles. Another study has also found that the resilient modulus significantly decreases within the first eight cycles, followed by a slightly decreasing amplitude [35]. Ceratti et al. revealed that the resilient modulus at the optimal moisture content decreases during up to the first four dry–wet cycles [36]. This is consistent with the findings in this paper. Cyclic dry–wet conditions will result in the breakdown and buildup of soil particles [37,38]. This is because of the irreversible swelling and the increase in saturation degree caused by the dry–wet cycle that lead to instability of interlocking structure and a softening behavior of the soil [39]. After that, the soil is more susceptible to yield. In addition, the smaller the deviator stress, the larger stable value of resilient modulus. The resilient modulus is about 66 MPa when the deviator stress is 30 kPa, while it is 52 MPa, 50 MPa, and 30 MPa at the deviator stress of 55 kPa, 75 kPa and 105 kPa, respectively.

### 4.3. Determination of Resilient Modulus Prediction Model

The testing data was used to calibrate the model coefficients involved in Equations (7) and (8), and the fitting parameters are presented in Table 4. The coefficient of determination values (*R*^2^) is larger than 0.9 for Equation (7), while the prediction model given by Equation (8) has a lower correlation. Thus, the final form of the resilient modulus prediction model in this paper is Equation (7).

## 5. FEM Analysis

### 5.1. Establishment of FEM Model

#### 5.1.1. FEM Model

**Figure 10:**
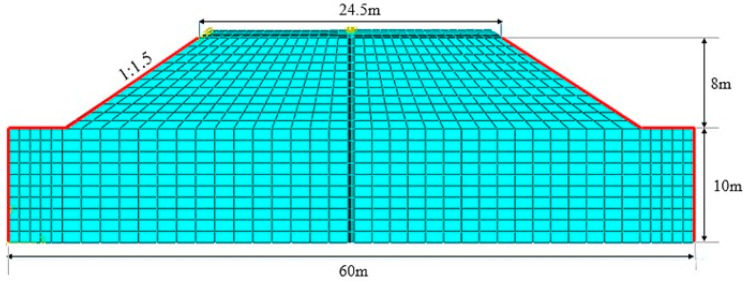
Finite element model of immersed subgrade.

The standard of a two-way four-lane highway and the design speed of 100 km/h were adopted. The section of the submerged subgrade is shown in Figure 10. The width of the top surface is 24.5 m. The overall pavement structure above the subgrade is 4 cm AC13 (asphalt concrete with a nominal maximum particle size of 13 mm), 6 cm AC20 (asphalt concrete with a nominal maximum particle size of 20 mm), 12 cm ATB25 (asphalt-treated base with a nominal maximum particle size of 25 mm), 16 cm continuously graded crush aggregate layer, and 32 cm cement stabilized gravel. The slope of subgrade was set to 1:1.5 [40], and the height to 8 m. The foundation size was 60 m × 10 m. The pavement structure was simplified as a plane strain. The typical plane strain CPE4 element with complete integral was used. As for the seasonal soaking subgrade, the subgrade and foundation should consider the coupling effect of the seepage field with the change in water level. Thus, the freedom degree of pore pressure should be considered. Finally, the completely integrated plane strain pore pressure element CPE4P was used. The two sides of subgrade and foundation were set as the water head boundary.

#### 5.1.2. Material Parameter

The linear elastic model was used in the numerical calculation of the pavement structure. The mechanical parameters that are needed in the modeling are elastic modulus *E*, Poisson’s ratio *μ* and material density *ρ*. The values of each parameter are determined based on the specifications of JTG D50-2017 [41], which are shown in Table 5.

In addition, since the subgrade and foundation are affected by the changes in water level, related hydraulics parameters such as the saturated permeability coefficient and corresponding reduction coefficient, and matrix suction are necessary for solving the fluid–solid coupling problem in FEM. The selection of the saturated permeability coefficient can refer to the empirical values of representative rock and soil listed in the specification of JTG/T D33-2012 [42]. The soil used in this paper is low liquid limit clay, and the saturated permeability coefficient is set as 5 × 10^−5^ mm/s. Considering that the variation in water level studied in this paper is in months, the permeability coefficient is converted to the value of 0.129 m/mon. The default calculation formula of the reduction coefficient in ABAQUS is shown in Equation (12), which is adopted in this paper. The volumetric water content could be converted to the saturation degree. Next, the values of matric suction and volumetric water content were calculated according to the SWCC. It is worth noting that the negative pore pressure is generally used instead of the matrix suction in the FEM.

(12) $$k_{s}={\left(S_{r}\right)}^{3}$$

Here, *k_s_* is the permeability reduction coefficient of unsaturated soil; and *S_r_*, the saturation degree.

#### 5.1.3. Periodic Change in Water Level

**Figure 11:**
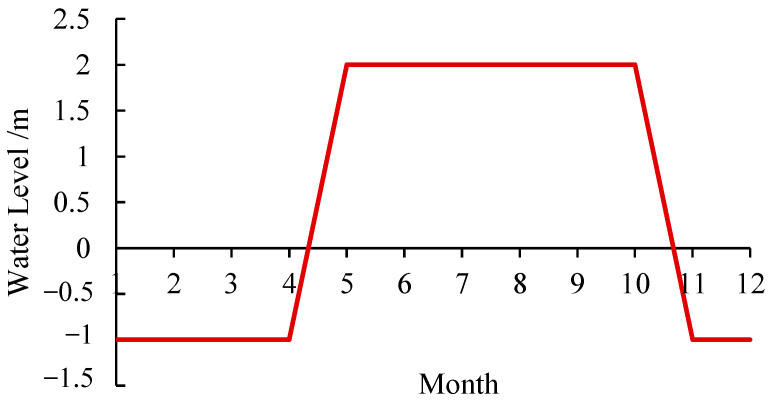
Annual water level change diagram.

The local rainfall is mainly concentrated from May to October, showing the characteristics of a long cycle and large rainfall. The rainy season and water depth are simplified for simulation calculations. Assuming that the initial groundwater level is located at −1 m, the rainfall starts from April to May and then continues until October, and the groundwater level rises to the maximum value, which is located at 2 m above the surface of the earth. As the rainfall weakens, the groundwater level begins to fall back to the initial groundwater level and remains unchanged. The variation in the annual water level of the subgrade is shown in Figure 11.

#### 5.1.4. Load and Mesh

The standard axle load of 0.7 MPa and loading radius of 0.213 m were applied in the general mechanical analysis. Since the plane two-dimension FEM model was used in this paper, the load had to be converted based on the equivalence principle and was determined as 117.37 kPa/s. The haversine wave with 0.1 s load pulse and 0.9 s rest period was adopted which is consistent with that used in the dynamic triaxial test.

#### 5.1.5. Calculation Logic

**Figure 12:**
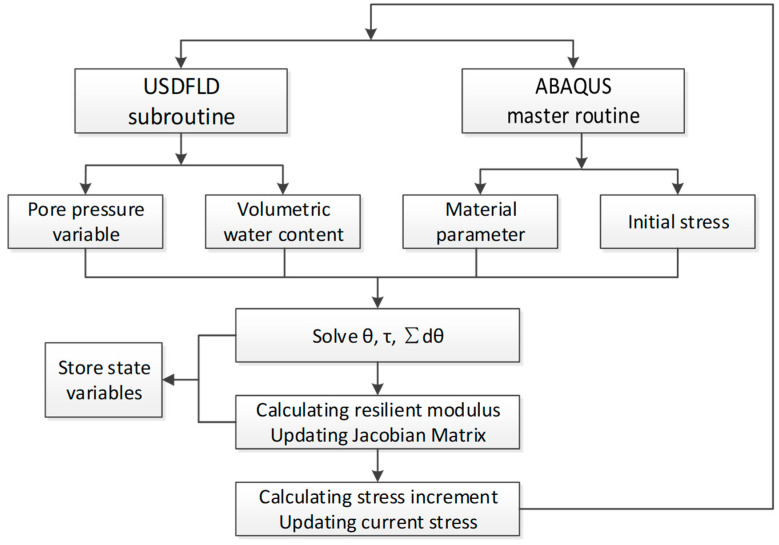
Algorithm flow chart of FEM.

The prediction model of resilient modulus requires hydraulic parameters such as the pore pressure and volumetric water content, which need to be obtained and stored as state variables before calling the UMAT subroutine. The USDFLD subroutine is used to redefine the field variables of the integration points. It is generally used with the built-in GETVRM subroutine to extract the output result data stored on the material integration points in the database. The calculation process for resilient modulus in ABAQUS is shown in Figure 12.

### 5.2. Result Analysis

#### 5.2.1. Analysis of Water Level Change

**Figure 13:**
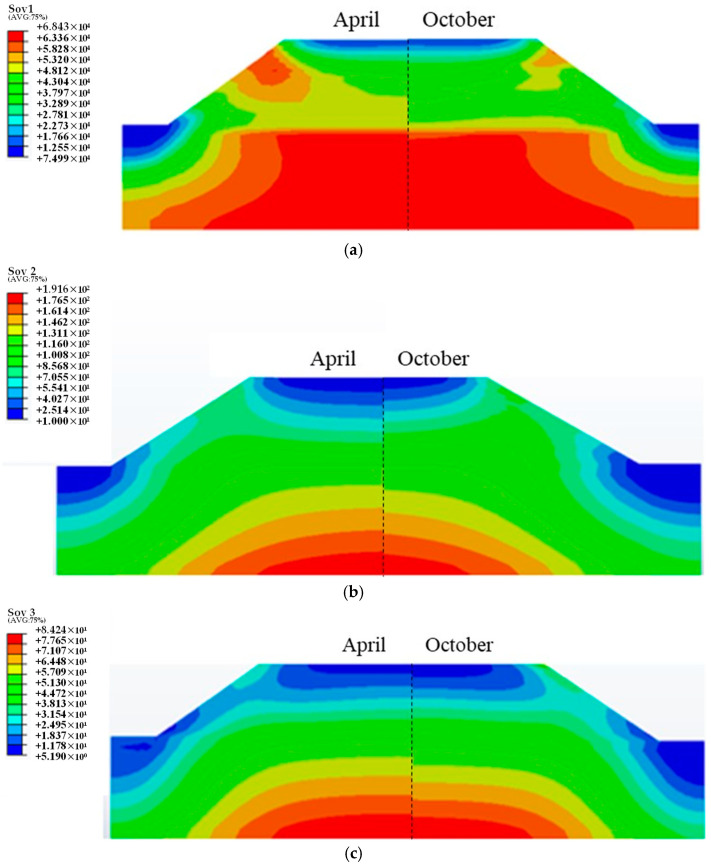
The mechanical response to soaking embankment: (**a**) resilient modulus, (**b**) bulk stress, (**c**) shear stress.

The cloud map of the distribution of resilient modulus was extracted for April when the water level started to rise, while October when the water level started to decline after a period of high-water level was used for comparison. Because of the symmetry of the model, half of the cloud maps of each characteristic month are shown. The resulting cloud map is shown in Figure 13.

The resilient modulus shows an increasing trend from the top surface to the inside. The modulus in April is higher than that in October. This is because the water level and the humidity are higher in October. The spatial distribution of bulk stress and shear stress is consistent with that of resilient modulus. This is because the upper part of the subgrade is less affected by the vehicle load compared to the gravity of subgrade. Thus, the minimum values of bulk stress and shear stress appear in the upper part of the subgrade. It can be seen from the constitutive model that the resilient modulus is positively correlated with the bulk stress and negatively correlated with the shear stress, that is, the two have opposite effects. Although the shear stress at the depth of the foundation is at the maximum value, the resilient modulus here is also the maximum value, which also shows the prominent role of volume stress in the calculation of resilient modulus.

#### 5.2.2. Analysis of Water Level Fluctuation

**Figure 14:**
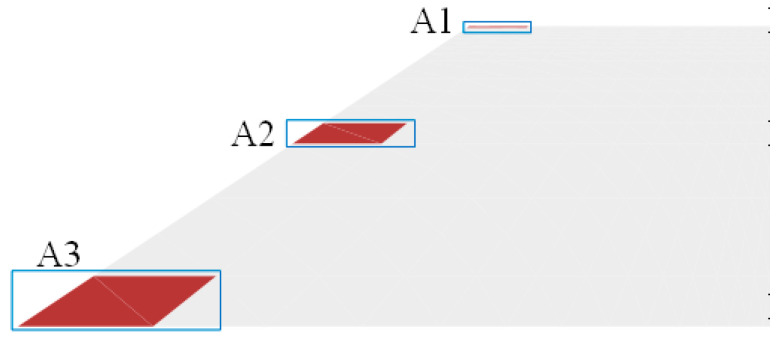
Selection of characteristic element.

**Figure 15:**
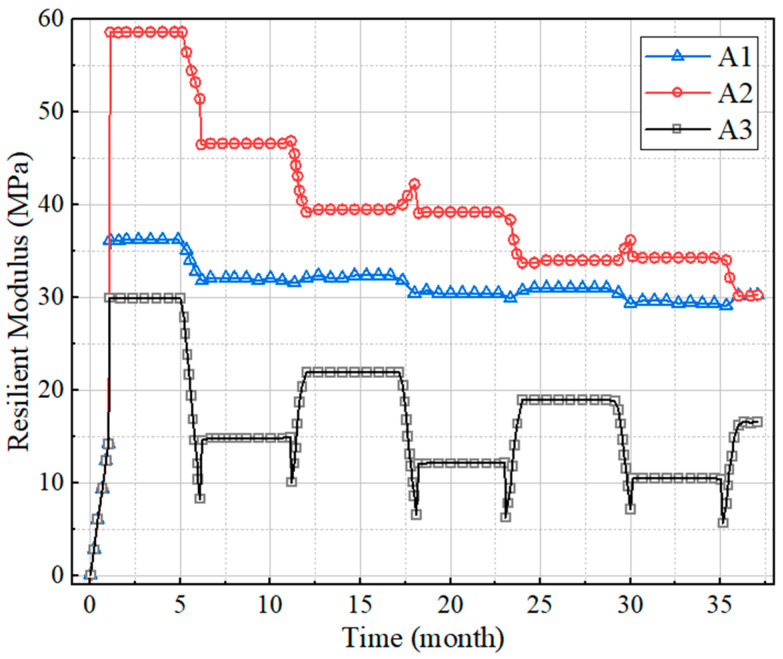
The relationship between resilient modulus and time.

**Figure 16:**
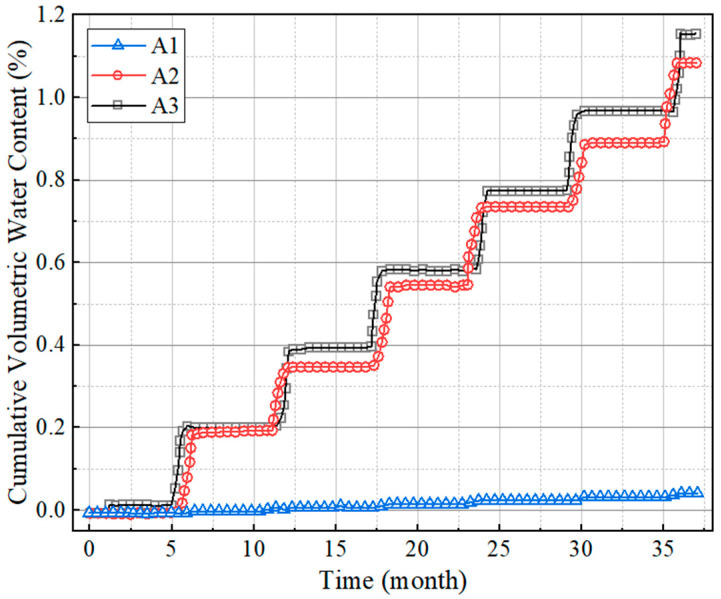
The relationship between cumulative volumetric water content and time.

The three elements A1, A2 and A3 were selected to represent the three positions of the upper, middle, and lower parts of the slope, respectively, as shown in Figure 14, and the variation in the bearing capacity of subgrade with time under the dry–wet cycle was studied. The resilient modulus and cumulative volumetric water content at three positions were extracted, and the time–history curves were drawn as shown in Figure 15 and Figure 16. It can be found that the resilient modulus in the middle and bottom of the slope decline significantly, and the initial resilient modulus at the position of A2 is the largest, at about 60 MPa. In the first year of dry–wet cycle, the decrease in the resilient modulus is the most severe, with a decrease of up to 18 MPa. For the top surface of the subgrade, the effect of water level changes is relatively small, with a decreasing amplitude of only 5 MPa during the three-year dry–wet cycle. This is closely correlated with the changes in cumulative volumetric water content in Figure 15. The cumulative volumetric water content at the positions of A2 and A3 increases with time, thus the resilient modulus decreases accordingly.

#### 5.2.3. Analysis of Monthly Equivalent Resilient Modulus

**Figure 17:**
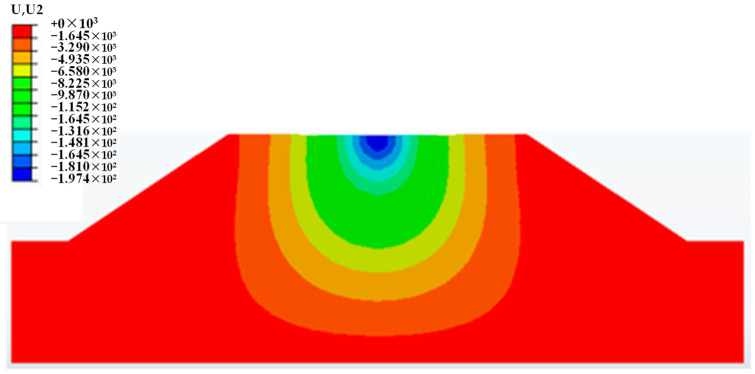
Vertical deformation of subgrade in October.

The weighted average equivalent method was adopted based on the transfer mechanism of vehicle load. The load is larger near the load position, and the contribution to the equivalent resilient modulus at the upper position should be larger than that at the lower position. The index of displacement distribution along the vertical direction of pavement structure was selected to use as a weight function. The vertical deformation of subgrade in October is shown in Figure 17. It can be seen that the vertical deformation of subgrade spreads downward from the load position. With the increase in depth, the deformation gradually decreases. This distribution law is consistent with the modulus weight of each part of the subgrade, that is, the position with large deformation should also display large modulus to resist the external load. Thus, the spatial distribution of vertical deformation inside the subgrade meets the requirements of weight function. In specific terms, the resilient modulus and maximum vertical deformation of all elements within the range of load influence were exported, and the value of equivalent modulus was then calculated according to Equation (13):

(13) $$E_{eq}=\frac{\sum M_{i}\cdot u_{i}}{\sum u_{i}}$$

where *M_i_* is the resilient modulus of each element; and *u_i_*, the vertical displacement of each element.

**Figure 18:**
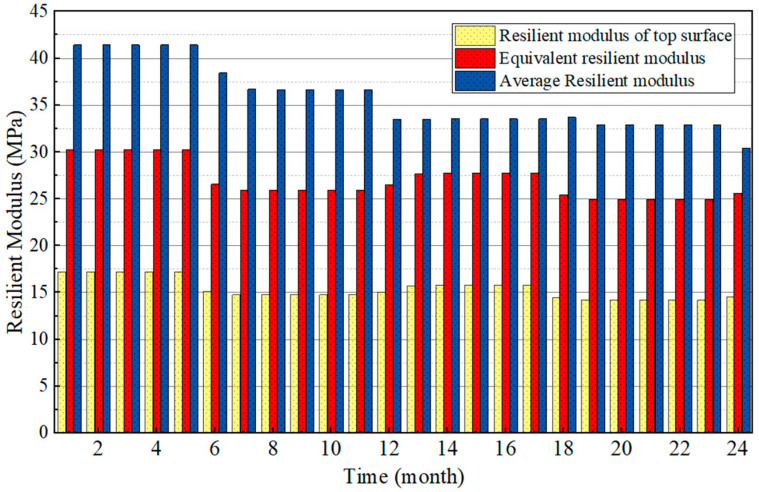
Comparison diagram of each equivalent resilient modulus.

The monthly equivalent resilient modulus of the subgrade was calculated and compared with the average resilient modulus and the resilient modulus of top surface, which are shown in Figure 18. The comparison shows that the fluctuation of three equivalent modulus values is the same, and varies in accordance with the change in water level. The subgrade in October is affected by the rise in the water level in the previous months and the continuous high-water level, resulting in larger humidity and lower resilient modulus compared with other months in the same year. When the water level then begins to decline, the resilient modulus increases. In addition, the three kinds of resilient modulus all show periodic changes during the two years and weaken over time until eventually stable. This is consistent with the simulation results of water level changes of representative elements. The average modulus is maximum, followed by the equivalent resilient modulus, while the resilient modulus of the top surface is minimum. The pavement is assumed to bear the same force at all positions when calculating the average modulus of the subgrade, while the weakening effect of load along the depth is not considered, which causes the modulus to be larger. The method for calculating equivalent resilient modulus is more in accordance with the actual stress condition of the subgrade. Therefore, it is more reasonable to use the equivalent resilient modulus to guide the design of subgrade.

## 6. Conclusions

This paper aimed to develop a new prediction model of resilient modulus for analyzing the effect of the dry–wet cycle on the resilient modulus of polymer reinforced soil. This included a FEM analysis which was performed to analyze the actual stress state of the pavement, and studying the effects of dry–wet cycle. The following conclusions could be drawn:

(1) The increase in deviator stress could result in a reduction in resilient modulus; the decreasing rate at low deviator stress is larger than that at a high level; and the resilient modulus is linearly positively correlated with the confining pressure.

(2) The effect of a dry–wet cycle could reduce the resilient modulus, whereby the reducing amplitude caused by the first dry–wet cycle is the largest, followed by a gradually stabilising trend.

(3) The initial resilient modulus at the middle position of slope is the largest and it also has the largest decreasing amplitude in the first year of dry–wet cycle; moreover, the resilient modulus at the upper position has the most stable trend.

(4) The FEM results show that the method for calculating equivalent resilient modulus is more in accordance with the actual stress condition of the subgrade, which is recommended to guide the design of the soaked subgrade.

## Figures and Tables

**Table 1:** Tested results of polymer-based reinforcement agent.

Tested Item	Testing Standards	Reference Standards	Tested Results
Density (g/cm^3^)	Soil stabilizing admixtures CJ/T 486-2015 [28]	D ± 0.03	1.41
pH		A ± 1.0	7.6
Soluble solids		S ± 2.0	96.5
Unconfined compression strength		≥2.5	3.2
Unconfined compressive strength ratio		≥120	148
Seven-day water stability coefficient ratio		≥105	115

Note: D, A and S denote the density, pH value and solid content of product control value, respectively.

**Table 2:** The physical indexes of soil.

Specific Gravity (g/cm^3^)	Maximum Dry Density (g/cm^3^)	Optimum Moisture Content (%)	Plastic Limit (%)	Liquid Limit (%)	Plasticity Index (%)
2.701	1.78	17.0	23.63	35.29	11.66

**Table 3:** Loading sequence of dynamic triaxial test.

Sequence Number	Confining Stress σ_3_ (kPa)	Deviator Stress σ_d_ (kPa)	$\mathrm{Vertical}\;\mathrm{Stress}\;\sigma_{1}$ (kPa)	Load Times/s
Pre-loading	30	55	85	1000
1	60	30	90	100
2	45	30	75	100
3	30	30	60	100
4	15	30	45	100
5	60	55	115	100
6	45	55	100	100
7	30	55	85	100
8	15	55	70	100
9	60	75	135	100
10	45	75	120	100
11	30	75	105	100
12	15	75	90	100
13	60	105	165	100
14	45	105	150	100
15	30	105	135	100
16	15	105	120	100

**Table 4:** Fitting results of prediction model.

Equation	*q*_1_	*q*_2_	*q*_3_	*q*_4_	*q*_5_	*R* ^2^
(7)	2340.93	0.40	−2.43	−0.61	0.28	0.93
(8)	1123.89	0.41	−2.49	0.99	−6.09	0.78

**Table 5:** FEM input parameters of pavement material.

Layer	Material Type	*E* (MPa)	*μ*	*ρ* (g/cm^3^)
Surface	AC13	11,000	0.25	2.42
	AC20	12,000	0.25	2.42
	ATB25	9000	0.25	2.42
Base	Graded crush aggregate	500	0.35	2.36
Subbase	Cement stabilized gravel	9000	0.25	2.35
Subgrade Foundation	Soil	60	0.4	1.83

## Data Availability

The data presented in this study are available on request from the corresponding author.
